# Risk Factors for Bunyavirus-Associated Severe Fever with Thrombocytopenia Syndrome, China

**DOI:** 10.1371/journal.pntd.0003267

**Published:** 2014-10-16

**Authors:** Fan Ding, Xu-Hua Guan, Kai Kang, Shu-Jun Ding, Li-Yong Huang, Xue-Sen Xing, Sha Sha, Li Liu, Xian-Jun Wang, Xiao-Mei Zhang, Ai-Guo You, Yan-Hua Du, Hang Zhou, Sirenda Vong, Xiao-Dong Zhang, Zi-Jian Feng, Wei-Zhong Yang, Qun Li, Wen-Wu Yin

**Affiliations:** 1 Chinese Center for Disease Control and Prevention, Beijing, P.R. China; 2 Institute of Infectious Disease Control and Prevention, Hubei Provincial Center for Disease Control and Prevention, Hubei, P.R. China; 3 Institute of infection Disease Control and Prevention, Henan Provincial Center for Disease Control and Prevention, Zhengzhou, P.R. China; 4 Shandong Center for Disease Control and Prevention, Shandong Provincial Key Laboratory of Communicable Disease Control and Prevention, Jinan, China; 5 Beijing Chaoyang District Center for Disease Control and Prevention, Beijing, China; 6 Division of Infectious Disease, Key Laboratory of Surveillance and Early-warning on Infectious Disease, Chinese Center for Disease Control and Prevention, Beijing, China; 7 World Health Organization (WHO), Representative Office in China, Beijing, China; University of Texas Medical Branch, United States of America

## Abstract

**Background:**

Severe fever with thrombocytopenia syndrome (SFTS) is an emerging disease that is caused by a novel bunyavirus, referred to as SFTS virus. During January 2011 to December 2011 we conducted a case-control study in Henan, Hubei and Shandong Provinces of China to determine the risk factors for SFTS.

**Methods:**

Case-patients were identified in hospitals and reported to provincial Centers for Disease Control and Prevention while being notified electronically to the National Surveillance System. Controls were randomly selected from a pool of patients admitted to the same hospital ward within one week of the inclusion of the cases. They were matched by age (+/−5 years) and gender.

**Results:**

A total of 422 patients participated in the study including 134 cases and 288 matched controls. The median age of the cases was 58.8 years, ranging from 47.6 to 70.1 years; 54.5% were male. No differences in demographics were observed between cases and controls; however, farmers were frequent and more common among cases (88.8%) than controls (58.7%). In multivariate analysis, the odds for SFTS was 2.4∼4.5 fold higher with patients who reported tick bites or presence of tick in the living area. Other independent risk factors included cat or cattle ownership and reported presence of weeds and shrubs in the working environment.

**Conclusions:**

Our findings support the hypothesis that ticks are important vectors of SFTS virus. Further investigations are warranted to understand the detailed modes of transmission of SFTS virus while vector management, education on tick bites prevention and personal hygiene management should be implemented for high-risk groups in high incidence areas.

## Author Summary

Since 2009, an emerging infectious disease which was identified as the severe fever with thrombocytopenia syndrome (SFTS) was reported in rural areas of Hubei, Shandong and Henan provinces in China. A novel bunyavirus designated severe fever with thrombocytopenia syndrome bunyavirus (SFTSV) had been identified to be the etiological cause of SFTS. But what risk factors lead to the disease is still not clear. Further investigations for risk factors are needed to effectively prevent and control the disease. Here we have designed case-control study to try to develop the risk factors of the spread of SFTSV. It is hoped that our research could provide epidemiological evidence for further study. Also help to determine the spread of the virus in the environment.

## Introduction

Severe fever with thrombocytopenia syndrome (SFTS) is an emerging disease that is caused by a novel bunyavirus, as referred to as SFTS virus (SFTSV) [1]. To date, the disease has only been reported in mainland China, Japan and Korea [2], [3] and which is characterized by high fever, thrombocytopenia, leukopenia, elevated serum hepatic enzyme levels, bleeding and multi-organ dysfunction and has an estimated case-fatality rate of 12% [4], [5]. The clinical manifestations can barely be differentiated from hemorrhagic fever with renal syndrome caused by hantavirus or human anaplasmosis [6], [7].

SFTSV is classified as a member of genus Phlebovirus, and thought to be transmitted by ticks as the virus has been isolated in *Haemaphysalis longicornis* ticks [1]. On a few account, the disease was also reported to transmit from person to person through contact with infected patient's blood or mucous [5], [6]. Real time RT- PCR and ELISA IgM are considered the assays of choice for early detection of SFTSV [8], [9].

In China, since 2010 SFTS has been a notifiable disease that should be reported within 24 hours as indicated by the National Notifiable Disease Surveillance System (NNDSS) and the national guideline for prevention and control of severe fever with thrombocytopenia syndrome (2010 edition) [10]. To date, cases have been reported in rural in the east and central part of China. The largest number of reported cases were in Henan, Hubei and Shandong provinces [11], [12]. During 2010–2011 most (75%) SFTS cases in China occurred yearly between May and August. Patients aged from 1 to 90 years (median, 58 years) and most were farmers (81%) including agricultural and forest workers from rural areas [13], [14].

Until now, we have not found any vector competence studies performed for SFTSV. The mode of SFTSV transmission remains unclear [15]–[17], and further investigations for risk factors are needed to effectively prevent and control the disease. Here we reported the first results of such a study which was conducted during January 2011 to December 2011 in Henan, Hubei and Shandong Provinces, China.

## Methods

### Ethics Statement

Informed consent was obtained from all study subjects prior to participation. All participants' personal identifiers were anonymized for confidentiality before China CDC received the dataset for pooled analysis. The study was approved by the Human Bioethics Committee, China CDC.

### Study Design and Setting

The Chinese Center for Disease Control and Prevention (China CDC) collaborated with provincial CDCs of Hubei, Henan and Shandong to design and conduct a risk factors-related case control study in affected areas. Cases were detected in hospitals and reported to provincial CDCs while being reported electronically to the NNDSS. Controls were randomly selected from a pool of patients admitted in the same hospital ward within one week of the inclusion of the cases. They were matched by age (+/−5 years) and gender regardless of symptoms (Tables 1 & 2).

**Table 1:** Baseline information regarding study sites, sample size and methods, Henan, Hubei and Shandong provinces, China, 2011.

ITEM	HuBei	HeNan	ShanDong	Total
**Hospital types** *				
Primary hospital	2	0	0	2
Secondary Hospital	13	3	2	18
Tertiary hospital	2	0	1	3
**Total**	17	3	3	23
**Field investigation**				
No. of study counties	7	3	3	13
No. of Study Participants	174	128	120	422
Matching Ratio	1∶2	1∶3	1∶3	-

*Hospitals are divided into three levels according to their functions (specialties) and technical capacities. Tertiary hospitals are the highest level.

**Table 2:** Laboratory testing results of the SFTS patients (cases, N = 134).

Testing	HuBei	HeNan	ShanDong	Total
PCR(+)alone	29	10	7	46
Ig M (+) alone	1	4	17	22
PCR(+) & IgM(+)	28	21	17	66
**Total**	58	35	41	134

When prodromal symptoms were present, the investigators of the local CDC would go to drawn the suspected case's blood. In the meanwhile, they would also search for the control for blood according to the matching requirement. Then the blood sample would be further confirmed in the labs.

### Case Definitions and Selection of Cases and Controls

As stated in the National guideline for prevention and control of severe fever with thrombocytopenia syndrome (2010 edition) [7], an SFTS case is defined as a patient who presents with fever (temperature is ≥38°C) associated with thrombocytopenia and leukopenia and subsequently requires testing for SFTSV. In this study, case subjects were defined as SFTS patients who had positive real-time RT-PCR for SFTSV or positive for IgM ELISA [1].

Control subjects were defined as matched patients whose laboratory testing for SFTSV infection (i.e. RT-PCR, IgM and IgG ELISA during the acute phase) was negative.

### Microbiological Analyses

The clinically diagnosed cases and controls' sera which collected by provincial CDC were separated from blood, aliquoted and transported in a cold box (4°C–8°C) to provincial CDCs' laboratories for testing. Confirmation consisted of real-time RT-PCR assay for SFTSV RNA and ELISA serology for IgM antibodies against SFTSV. All diagnostic kits were provided by the National Institute for Viral Disease Control and Prevention of China CDC in Beijing and laboratory technicians received refresher courses regarding testing techniques.

### Data Collection and Storage

Fifty professionals from China CDC, provincial and county CDCs were trained to interview and administer a standardized questionnaire to cases and controls. About 70 clinicians across primary, secondary and tertiary hospitals were also trained on case reporting. These clinicians also accepted differential diagnoses refresher courses for SFTS. Participants/patients were asked about their demographics (age, gender, ethnic group, home address, occupation), living environment (e.g. landform, environment, poultry, animal raising, house rats, wild animals), exposure history within the previous 2 weeks prior to fever onset (e.g. travel history, tick bites, contact with suspected SFTS patients, contact with similar cases) and contacts with animals (animal species and types of vectors).

Completed questionnaires were systematically verified by China CDC study coordinators for data completeness. Data were double-entered into an Epidata 3.02 (the EpiData Association, Denmark, Europe) database followed by consistency checking.

### Statistical Analysis

SPSS version 18.0 (Statistical Product and Service Solutions, Chicago, IL, USA) was used for all statistical analyses. All tests were 2-tailed; statistical significance was set at *P*<0.05, without correction for the number of statistical tests performed. We compared proportions with use of Pearson Chi-Square and Fisher's exact test. In multivariate analysis, maximum likelihood estimates for the matched odds ratios (ORs) were calculated using a conditional logistic regression model and the Wald test. We retained in the model the significant variables (P<0.05).

## Results

A total of 422 persons participated in the study including 134 cases and 288 matched controls. And 13 counties and 23 hospitals took part in the research with no refusals. The matching ratio is about 1∶3. On PCR testing, only 46 cases had positive results. And 22 cases' IgM ELISA results were positive.

The median age of the cases was 58.8 years (range, 47.6∼70.1 years) and 54.5% were male. No differences in demographics (e.g. gender, ethnicity and residence) were observed between cases and controls; however, farmers were frequent and more common among cases (88.8%) than controls (58.7%). All of the cases' ethnicity were Han.

As shown in the univariate analysis, potential risk factors for SFTS included those associated with ownership or contact with domestic animals, presence of rats in the households, potential exposure to wild animals, indirect or direct exposure to ticks, and contact with potentially contaminated environment. Details are presented in table 3.

**Table 3:** Variables associated with SFTS, univariate analysis, central and eastern China, 2011.

Factor	Case group (*n* = 134)	Control group (*n* = 288)	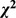	*P value*	OR
**Gender**					
Male	73(54.5%)	160(55. 6%)	0.1	0.8	0.9
Female	61(45.5%)	128(44.4%)			
**Age range in years**	47.6∼70.1	48.3∼70.4	-	-	**-**
**Residence**					
Rural	134(100.0%)	284(98.6%)	0.7	0.4	1.0
Urban	0(0)	4(1.4%)			
**Ethnicity**					
Han	134(100.0%)	284(98.6%)	0.7	0.4	1.0
Other	0(0)	4(1.4%)			
**Occupation**					
Farmer	119(88.8%)	169(58.7%)	38.3	<0.01	1.5
Other	15(11.2)	119(41.3%)			
**Patients among relatives or neighbors** *	2(1.5%)	5(1.7%)	0.1	0.8	0.9
**Leaving place in the previous month** *	7(5.2%)	26(9.0%)	1.8	0.2	0.6
**Domestic animals**					
**Owned dogs**	70(52.2%)	107(37.2%)	8.6	<0.01	1.9
Captive or not*	63(90.0%)	71(66.4%)	12.9	<0.01	4.6
Presence of tick on dogs	26(37.1%)	21(19.6%)	6.7	<0.01	2.4
Contact with a dog 2 weeks prior to disease onset	49(70.0%)	43(40.2%)	15.1	<0.01	3.5
**Owned cats**	41(30.6%)	32(11.1%)	24.3	<0.01	3.5
Free-roaming or not* #	38(92.7%)	26(81.3%)	1.2	0.3	2.9
Presence of ticks on cats#	3(7.3%)	4(12.5%)	0.1	0.7	0.6
Contact with a cat 2 weeks prior to disease onset	24(58.5%)	7(21.9%)	9. 9	<0.01	5.0
**Owned cattle**	48(35.8%)	32(11.1%)	36.3	<0.01	4.5
Been guarded or not* #	43(89.9%)	28(87.5%)	0.0	0.9	1.2
Presence of tick on cattle	28(58.3%)	14(43.8%)	1.6	0.2	1.8
Contact with a cattle 2 weeks prior to disease onset	33(68.8%)	24(75.0%)	0.4	0.6	0.7
**Owned goats**	12(9.0%)	16(5. 6%)	1.7	0.2	1.7
**Owned pigs**	40(29.9%)	47(16.3%)	10.2	<0.01	2.2
Captive or not* #	3(7.3%)	7(15.9%)	0.8	0.4	0.4
Presence of tick on the pigs#	2(5.0%)	2(4.7%)	0.1	0.7	1.2
Contacting with a pig 2 weeks prior to disease onset	25(62.5%)	19(40.4%)	4.2	<0.05	2.5
**Poultry**	53(39.6%)	63(21.9%)	14.3	<0.01	2.3
Captive or not*	45(84.9%)	45(71.4%)	3.0	0.9	2.3
Presence of tick on poultry's hide#	4(7.6%)	5(7.9%)	0.1	0.8	1.0
Contact with poultry 2 weeks prior to disease onset	28(52.8%)	17(27.0%)	8.1	<0.01	3.0
**Rodents**					
Presence of rats in home	92(68.7%)	148(51.4%)	11.1	<0.01	2.0
Touched a rat	6(6.5%)	26(17.0%)	5.6	<0.05	0.3
Touched a dead rat	4(3.0%)	7(2.4%)	0.0	1.0	1.2
**Wild animals in the surroundings of household**					
Presence of wild animals	72(53.7%)	109(37.9%)	9.4	<0.01	1.9
Presence of voles	53(74.6%)	87(78.4%)	0.3	0.6	0.8
Presence of hares	58(81.7%)	77(69.4%)	3.4	0.1	2.0
Muntjacs	2(2.9%)	0(0%)	-	0.2	-
Presence of boars	35(48.6%)	32(28.8%)	7.4	<0.05	2.3
Pheasants	50(69.4%)	63(56.7%)	3.0	0.1	1.7
Weasels	33(46.5%)	48(43.2%)	0.2	0.7	1.1
Owls	25(35.2%)	24(21.6%)	4.1	<0.05	2.0
Snake	56(78.9%)	64(57.7%)	8.7	<0.01	2.7
Hedgehogs	33(47.8%)	41(37.3%)	2.0	0.2	1.5
**Practice hunting** # *	4(3.0%)	2(0.7%)	2.0	0.2	4.4
Direct contact with wild animals 2 weeks prior to disease onset	6(4.5%)	1(0.4%)	9.7	<0.01	13.6
**Ticks**					
Presence of ticks in households	75(56.0%)	62(21.5%)	49.5	<0.01	4.6
Removed ticks from domestic animals 2 weeks prior to disease onset	19(14.2%)	14(4.9%)	11.0	<0.01	3.2
Tick bites 2 weeks prior to disease onset	20(14.9%)	6(2.1%)	26.1	<0.01	8.3
**Environment**					
Worked in the field	100(74.6%)	120(41.8%)	39.4	<0.01	4.1
Worked in hill areas	69(69.0%)	72(70.0%)	1.9	0.2	1.5
Presence of rats in the field#	37(27.6%)	31(25.8%)	3.2	0.1	1.7
Tea harvesters	19(14.2%)	24(8.3%)	3.4	0.1	1.8
Presence of weeds and shrubs in working areas	95(73.6%)	114(44.4%)	29.7	<0.01	3.5
Working with protective equipment	13(9.7%)	18(6.3%)	1.6	0.2	1.6
Skin bruises from field work	25(18.7%)	23(8.0%)	10.3	<0.01	2.6
Sit on the grass during work break	65(48.5%)	70(24.3%)	24.6	<0.01	2.9
Other working pattern*	21(15.7%)	8(2.8%)	24.8	<0.01	6.7
Presence of weeds and shrubs around household	104(77.6%)	132(45.8%)	37.5	<0.01	4.1

# means Fisher's Exact Test outcome.

* “Patients among relative or neighbors” means the relative or neighbors of subject of the investigation who may surffer from SFTS.

“leaving place in previous month” means long-distance travel.

“Captive or not” means whether raising animals as pets or feeding animals in a pen.

“Free-roaming or not” means whether cage free.

“Been guarded or not” means whether raising cattles in the cowshed.

“Practice hunting” means hunting is just one's hobby not live by that.

“Other working pattern” means the grazing, hunting and other pattern to work.

In the multivariate analysis, the odds ratio for SFTS was 2.4 to 4.5 fold higher with patients who reported tick bites or presence of ticks in the living area. Other independent risk factors included cat or cattle ownership and, reported presence of weeds and shrubs in the working environment (Table 4).

**Table 4:** Factors associated with SFTS, multivariate analysis, central and eastern China, 2011.

Factors	Cases (n = 134)	Controls (n = 288)	*P value*	OR	95% *CI* (Low-Up)
Tick bites 2 weeks prior to disease onset	20(14.9%)	6(2.1%)	<0.01	4.5	1.6–12.9
Owned cattle	48(35.8%)	32(11.1%)	<0.01	2.6	1.4–4.8
Presence of ticks in living area	75(56.0%)	62(21.5%)	<0.01	2.4	1.4–4.0
Worked in the field	100(74.6%)	120(41.8%)	<0.01	2.3	1.3–4.0
Owned cats	41(30.6%)	32(11.1%)	0.02	2.13	1.2–3.9
Presence of weeds and shrubs in working areas (outdoor work)	95(73.6%)	114(44.4%)	0.04	1.91	1.0–3.5

## Discussion

As suggested by initial investigations, association with ticks appear to be a major risk factor for acquiring SFTS in our study. The odds ratio for report of tick bites two weeks prior to SFTS onset was the highest (∼5) of all the identified independent risk factors; however tick bites was only reported by about 15% of the cases. As these bites were commonly painless, it is possible that the bites might have gone unnoticed among many patients, hence underestimating the number of tick bites reports. The majority of individuals with these bites develop no symptoms and many do not remember getting bitten [1], [4]–[6]. Other identified risk factors such as presence of ticks in living areas, outdoor and field work or cats/cattle ownership also converged to suggesting that ticks are the main vector of transmission. Peasants and individuals are commonly inclined to touch the hide of these cats or cattle, exposing them to potential tick bites. Previous studies have detected SFTSV in *Haemaphysalis longicornis* and *Rhipicephalus microplus* ticks collected from several domestic animals, including cattle, buffaloes, goats, cats and dogs and serology positive for IgM antibodies against SFTSV was observed in dogs, cattle and other livestock in villages where the patients lived [1], [12], [18], [19]. *Haemaphysalis spp.* are commonly found in humid scrubby forests, meadows or peatlands; a natural environment that is consistent with mountain or hilly areas of our studied provinces. Most *Haemaphysalis* species are three-host ticks, i.e. can be found free-living in the environment waiting for a suitable host (e.g. small mammals but also reptiles and birds, domestic animals and wildlife). *Haemaphysalis longicornis* in particular is also known as bush or scrub tick and occurs in other countries in the Western Pacific Rim. Nevertheless, further ecological investigations are needed to determine the range of potential vectors among the ticks group. Our study covered most cases of three provinces which has important public health significance. Until now, no researches can complete so deeply or fail to cover such large area in China. Their results are only applicable to local areas. But we offered a comprehensive field investigation. The focus of our research is to detect and identify the various risk factors (e.g. environment, working conditions, host and so on) of SFTS by field epidemiology.

Given that person-to-person transmission may be possible as reported in a family cluster in Jiangsu province, China [5], [6], our finding suggests that person-to-person may not be common as none of our cases reported any close contacts (i.e. relatives) that were sick during a 2-week potential incubation period. However, this speculation would only be better understood when one further investigates on the extent of the clinical spectrum of SFTSV infected individuals, including the extent of subclinical manifestations and the proportion of asymptomatic cases.

The findings in the present study need to be interpreted in light of some major limitations. Firstly, one fundamental selection/surveillance bias lies on the case definition of SFTS; as written, clinicians may be inclined to only report or think about SFTS when the patient presents with epidemiological links such as tick bites or residing in hilly areas. In addition, high media attention was drawn into these provinces when the virus was discovered in 2009–2010. In short, clinicians may distort their perceptions that patients with SFTS who resided in the plains or urban areas would not be at-risk for SFTS, hence, excluding explorations of risk factors in these particular areas. Secondly, biases could have occurred by only selecting controls from hospitals. These controls may have not been drawn from the same environment (e.g. they may only reside in low risk areas), which would have overpowered transmission risks associated with ticks or outdoor activities. Unfortunately the place of residence for cases and control was not collected. However, this variable may have been indirectly controlled in our analysis because most selected study hospitals were located in hilly areas. It is therefore likely that controls and cases also came from the same surroundings area of the study hospitals.

Finally, the SFTS cases detected in the study were middle or older-aged residents in hilly regions of the three studied hilly/rural areas; this age distribution was no different from that of surveillance derived cases. The reasons for this particular age distribution have not yet been fully addressed in initial reports, including the present study. We instead chose to control age with an age-matched case-control study to increase statistical power. Our common understanding was that the cases' age distribution may only reflect the age demographics of Chinese population in rural areas. Because a large number of young adults migrated from rural to urban areas, rural areas are left with an age distribution skewed towards seniors. Unfortunately population data for the study areas were not available to assess the extent to which this migration is affecting the population age structures between rural and urban China. A larger share of the population was less than 15 years of age, possibly due to relatively high fertility rate; however, few cases among this age-group were reported. For effective prevention measures, further investigations are warranted to understand whether this age pattern is related to at-risk behaviors, SFTS-related clinical spectrum, intensity of contact, weakened immunity or/and surveillance biases.

### Conclusion

This study was conducted during the early phase of the discovery of the virus and should be seen as a preliminary step within an in-depth investigation of risk factors for SFTS when it was urgent to confirm the role of ticks as the main vector of transmission. The detailed modes of transmission of SFTSV were not addressed in the study and warrants further investigations. Direct contacts with animals especially those that are free-roaming and participating in outdoor activities in dense vegetation areas increase opportunities for people to be bitten by ticks. Integrated vector management and ecosystems interventions should be implemented to reduce the density of ticks in working and living environments. Education on tick bites prevention and personal hygiene management are of utmost importance for high-risk groups in high incidence areas.

## Supporting Information

Checklist S1 STROBE checklist.
(DOC)
